# The association between variability of risk factors and complications in type 2 diabetes mellitus: a retrospective study

**DOI:** 10.1038/s41598-024-56777-w

**Published:** 2024-03-16

**Authors:** Mengjie Chen, Lihui Pu, Yuqin Gan, Xiaoxia Wang, Laixi Kong, Maoting Guo, Huiqi Yang, Zhe Li, Zhenzhen Xiong

**Affiliations:** 1 School of Nursing, Chengdu Medical College, No. 601 Tian Hui Road, Rong Du Avenue, Chengdu, 610083 Sichuan China; 2 Menzies Health Institute Queensland, Griffith University, Brisbane, QLD 4111 Australia; 3 Nanbu County People’s Hospital, Nanchong, 637300 Sichuan China; 4 Mental Health Center, West China Hospital, Sichuan University, No. 28 Dianxin South Road, Chengdu, 610041 Sichuan China; 5 Sichuan Clinical Medical Research Center for Mental Disorders, No. 28 Dianxin South Road, Chengdu, 610041 Sichuan China; 6 School of Nursing and Midwifery, Griffith University, Queensland, Australia; 7 Erasmus MC, University Medical Centre Rotterdam, Department Internal Medicine, Section Nursing Science, Rotterdam, The Netherlands

**Keywords:** Type 2 diabetes mellitus, Complications, Total cholesterol, Outpatient special disease management, Variability, Endocrinology, Risk factors

## Abstract

The variability in diabetes risk factors, such as uric acid and lipids, may influence the development of complications. This study aimed to investigate the influence of such variability on the occurrence of diabetic complications. A retrospective analysis of electronic medical records was conducted with type 2 diabetic patients who received treatment at a tertiary care hospital in Chengdu, Sichuan Province, between 2013 and 2022. The risk factor variability is presented as the standard deviation (SD). The associations between the variability and complications were examined using a binary logistic regression model. The study included 369 patients with type 2 diabetes. The findings revealed that outpatient special disease management served as a protective factor against the development of complications [OR = 0.53, 95% confidence interval (CI) (0.29–0.10)], particularly for the prevention of diabetic peripheral neuropathy [OR = 0.51, 95% CI (0.30–0.86)]. Variability in total cholesterol (TC-SD) was found to be a risk factor for the development of complications [OR = 2.42, 95% CI (1.18–4.97)] and acted as a risk factor for diabetic peripheral vasculopathy [OR = 2.50, 95% CI (1.25–5.02)]. TC-SD is a risk factor for the occurrence of diabetic peripheral neuropathy and diabetic peripheral vasculopathy, whereas outpatient special disease management functions as a protective factor against complications and diabetic peripheral neuropathy. Thus, in addition to glycaemic control, the regulation of lipid levels should be emphasized, particularly among patients without outpatient special disease management, to delay the onset of complications.

## Introduction

Due to factors such as the rapid growth of the domestic economy and increases in urbanization, the standard of living, and the ageing population, the prevalence of diabetes in China has been steadily increasing^1^. According to an epidemiological survey conducted by the Endocrinology Branch of the Chinese Medical Association on Diabetes between 2015 and 2017, the prevalence of diabetes among Chinese adults over the age of 18 was 11.2%, with approximately 90% of patients having type 2 diabetes mellitus (T2DM)^2^.

As a chronic condition, diabetes gradually progresses to complications that worsen with disease progression. These complications not only diminish patients' quality of life and increase medical expenses, treatment complexity, and mortality rates, but they also impose a significant socioeconomic burden^3^. In the United States, the cost of treating complications associated with T2DM constitutes 53% of the lifetime medical expenses for T2DM patients^4^. Several surveys that were conducted to assess the costs of diabetes treatment in several Asian countries have indicated that there is a positive correlation between the occurrence of complications in T2DM patients and the burden of treatment, and hospital expenditures for diabetic patients with complications are 2–3 times greater than those for patients without complications, suggesting that these patients consume substantial health care resources^5,6^.

Diabetic complications include both macrovascular and microvascular manifestations. Macrovascular complications primarily manifest as cardiovascular disease and peripheral vascular disease^7^, whereas microvascular complications mainly involve retinopathy, neuropathy, and nephropathy^8^. Diabetic peripheral neuropathy (DPN) represents the most prevalent microvascular complication in patients with diabetes mellitus^9^. DPN significantly contributes to lower-limb amputation and debilitating neuropathic pain^10^. Diabetic peripheral vasculopathy (DPV) is a prevailing chronic complication of diabetes mellitus. It represents a frequent cause of disability among individuals with diabetes and has detrimental effects on the digestive, neurological, and vascular systems as the disease progresses^11^. DPV significantly increases the risk of claudication, ischaemic ulcers, gangrene, and amputation^12^. Moreover, diabetic retinopathy (DR) poses visual and cardiovascular hazards to patients and is a leading cause of vision loss among elderly people^13^. It serves as a predictive indicator for all-cause mortality, vascular-related mortality, and noncancer mortality^14,15^. Furthermore, diabetic nephropathy is another prevalent complication. It is the leading cause of mortality in type 2 diabetes patients and is characterised by early manifestations of proteinuria and oedema and eventual progression to renal failure^16^. Deleterious vascular complications lead to a decline in overall quality of life and an increased mortality rate among individuals with diabetes mellitus. Therefore, controlling the occurrence and progression of type 2 diabetes complications represents a pivotal measure for enhancing patients' disease prognosis, improving their quality of life, and alleviating economic burdens on both families and society.

Current management approaches for diabetic patients primarily focus on medications to control glycaemic levels to mitigate the progression of complications^17^. A systematic review revealed^18^ that diabetic patients taking metformin had a lower risk of all-cause mortality, cardiovascular mortality, and cardiovascular disease occurrence. Additionally, other diabetes-related risk factors significantly contribute to the development of diabetic complications. For example, individuals with prediabetes commonly exhibit concurrent cardiovascular risk factors such as hypertension and dyslipidaemia, thereby augmenting their susceptibility to cardiovascular diseases^19,20^. Noteworthy risk factors associated with diabetes include lipid parameters (including total cholesterol, high-density lipoprotein, low-density lipoprotein, and triglyceride levels) and uric acid levels^21^. Dyslipidaemia, a prevailing risk factor for cardiovascular ailments, is the leading cause of mortality in individuals with both chronic kidney failure (CKD) and T2DM^22^. Studies have demonstrated elevated plasma concentrations of total cholesterol, triglycerides, and low-density lipoprotein in patients presenting with concurrent diabetic neuropathy, thereby further escalating their cardiovascular risk^23^. Moreover, renal function in diabetic patients gradually deteriorates with increasing uric acid levels, and greater levels of uric acid are associated with an increased risk of retinopathy^24,25^.

To increase the accuracy of predicting the occurrence of complications, scholars^26,27^ have posited the concept of glycaemic variability. Multi-country investigations^27–29^ have revealed associations of variability in haemoglobin A_1c_ (HbA_1c_) levels with all-cause mortality, renal disease, and cardiovascular disease among individuals diagnosed with T2DM. Like glycaemic variability, variations in diabetes risk factors may also be correlated with diabetic complications^21,30^. Numerous studies^31–33^ have shown a link between lipid variability and cardiovascular events as well as all-cause mortality in patients with T2DM. Furthermore, elevated uric acid levels serve as a risk factor for cardiovascular disease and kidney disease in diabetic patients^34^. While complications in diabetic patients are influenced by multiple factors, no study to date has concurrently explored the relationship between uric acid and lipid variability and complications. Therefore, this retrospective study aimed to investigate the impact of variable diabetic risk factors on complications and provide clinical evidence for delaying the onset and progression of such complications.

## Study methods

### Study design and participants

The data were collected from the outpatient clinic of the Department of Endocrinology in a tertiary care hospital located in Chengdu, China. The data collection spanned from 2013 to 2022 and included a total of 369 patients with T2DM. The inclusion criteria included ① adherence to the^35^ 1999 version of the diagnostic and classification criteria for T2DM established by the World Health Organization (WHO); ② age of 18 years or older; ③ absence of complications at baseline; and ④ completion of a minimum of 3 follow-up visits. Specific exclusion criteria can be found in our published article “A study of factors influencing long-term glycaemic variability in patients with type 2 diabetes: a structural equation modelling approach”^36^.

#### Diabetes complications

The diabetic complications investigated in this study included diabetic peripheral neuropathy, diabetic peripheral vasculopathy, diabetic nephropathy, and diabetic retinopathy. Complications were diagnosed based on the clinical diagnosis made by outpatient special physicians following standard diagnostic methods.

#### Risk factors for variability indicators

The risk factors included uric acid (UA) and lipids, the latter of which mainly comprise triglycerides (TGs), total cholesterol (TC), high-density lipoprotein (HDL), and low-density lipoprotein (LDL). The mean variability of the risk factors was defined as the average of all available measurements, while the risk factor variability was represented by the standard deviation (SD)^32^.

### Statistical analysis

The deidentified data were extracted from EpiData (Chinese version) management software, and the statistical analysis was conducted using SPSS 27.0 (IBM, Chicago, IL, USA). The SD served as a measure of risk factor variation and was divided by the square root of the ratio of ‘total visits’ to ‘total visits minus 1’ to adjust for the possible effect of the number of visits on variation^33^. Quantitative data are presented as the mean ± standard deviation ($\overline{\chi }$ ± SD), and nonnormally distributed data are presented as the median (IQR). Descriptive data are reported as n (%). Independent-samples t tests were used to compare two groups, analysis of variance (ANOVA) was used for comparing multiple groups, and Mann–Whitney U and Kruskal–Wallis tests were used for non-parametric data. The associations between the variability risk factors and diabetic complications were examined using a binary logistic regression model. P < 0.05 was considered to indicate statistical significance.

### Ethical considerations

This study was approved by the Ethics Committee of the First Affiliated Hospital of Chengdu Medical College (approval no. 2022CYFYIRB-BA-Dec01), and it was carried out in accordance with the Code of Ethics of the World Medical Association (Declaration of Helsinki).

### Need for and requirement of informed consent

The need for informed consent was waived by the ethical committee of The First Affiliated Hospital of Chengdu Medical College IRB.

## Results

### Clinical characteristics of study participants

Among the 369 participants with T2DM, the ages ranged from 30 to 97 years, with a mean of 61.92 ± 11.70 years. The follow-up time ranged from 1 to 10 years, with a mean of 5.14 ± 2.25 years. A total of 259 (70.2%) patients were managed for outpatient-specific diseases, and a total of 313 (84.8%) patients had complications. Moreover, 85.1% of patients experienced the simultaneous occurrence of two or more complications, 4.9% of diabetic patients being burdened by the coexistence of four distinct complications. The demographic information of the participants is shown in Table 1.

**Table 1:** Sociodemographic characteristics of the study subjects (n = 369).

	Category	Sample n (%)
Sex	Male	190 (51.5%)
	Female	179 (48.5%)
Age, years	≤ 60	175 (47.4%)
	> 60	194 (52.6%)
Follow-up time, years	1–3	112 (30.4%)
	4–6	123 (33.3%)
	≥ 7	134 (36.3%)
OSDM	Yes	259 (70.2%)
	No	110 (29.8%)
Complications	Yes	313 (84.8%)
	Diabetic peripheral neuropathy	285 (77.2%)
	Diabetic peripheral vasculopathy vasculopathy vasculopathy	231 (62.6%)
	Diabetic retinopathy	9 (2.4%)
	Diabetic nephropathy	6 (1.6%)
	No	56 (15.2%)
Number of complications*	1	46 (14.9%)
	2	166 (53.7%)
	3	82 (26.5%)
	4	15 (4.9%)

*OSDM* outpatient special disease management.

*Number of complications: the number of complications rather than the number of cases.

### Characteristics of the variable distribution of diabetes risk factors

**Figure 1:**
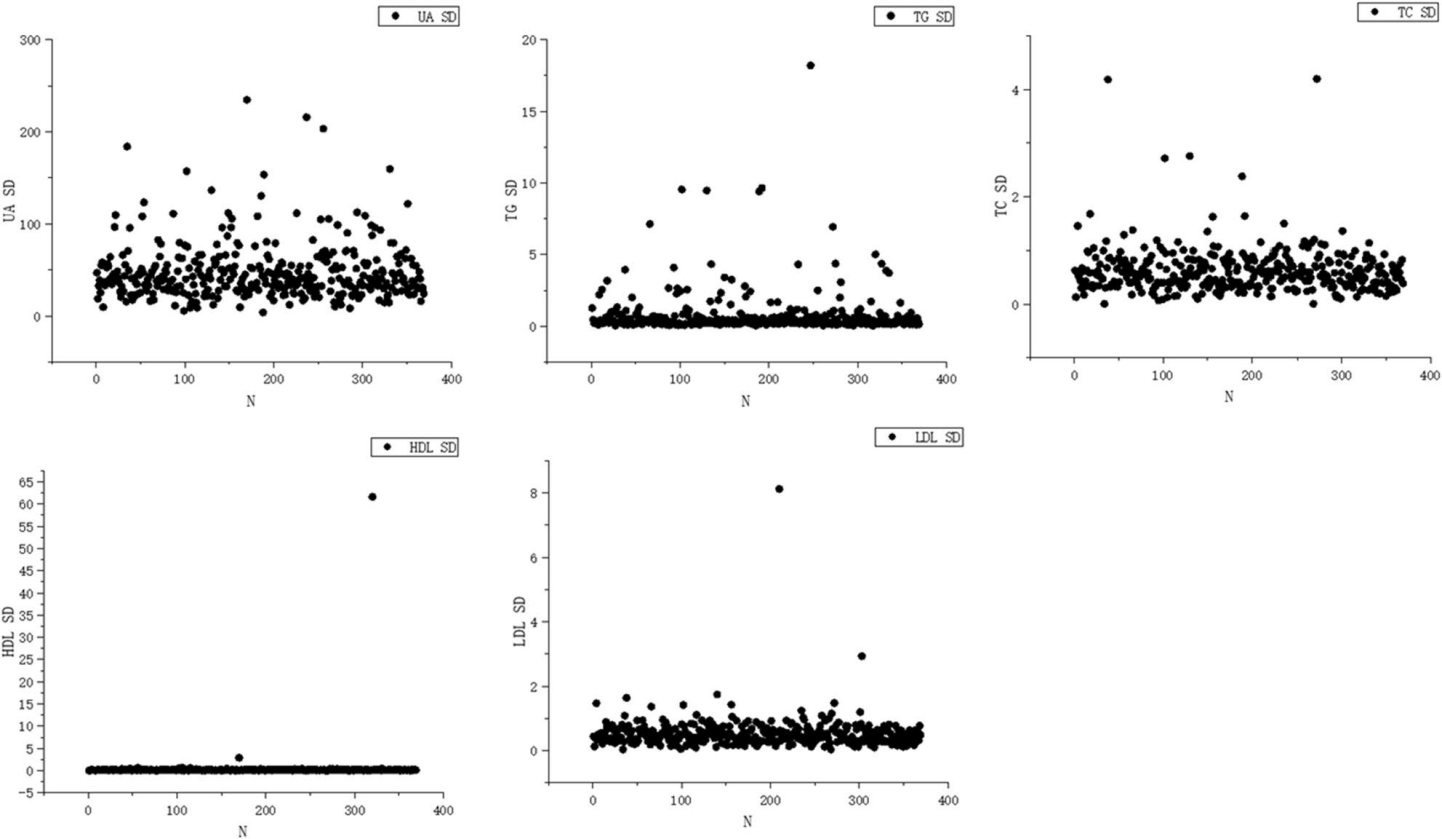
Variability in risk factors for type 2 diabetes mellitus.

In this study, triglycerides standard deviation (TG-SD), high-density lipoprotein standard deviation (HDL-SD) and low-density lipoprotein standard deviation (LDL-SD) were non-normally distributed, and were therefore expressed as median (IQR).The mean uric acid standard deviation (UA-SD) was 47.68 ± 30.74, the mean total cholesterol standard deviation (TC-SD) was 0.62 ± 0.44; the median TG-SD was 0.33 (0.20, 0.63), the median HDL-SD was 0.14 (0.10, 0.17), and the median LDL-SD was 0.47 (0.30, 0.65). The distributions of UA-SD, TG-SD, TC-SD, HDL-SD and LDL-SD are shown in Fig. 1.

### Univariate analysis of the variability in risk factors for diabetes mellitus

Univariate analysis revealed that the sex of diabetic patients had a statistically significant (*P* < 0.05) impact on UA-SD and HDL-SD, with higher UA-SD levels observed in males than in females, while females had higher HDL-SD levels than males did. Age was significantly (*P* < 0.05) associated with TG-SD and TC-SD, with patients aged 60 years and younger exhibiting greater TG-SD and TC-SD. The implementation of outpatient special disease management had a statistically significant (*P* < 0.05) influence on HDL-SD, with patients who did not receive outpatient special disease management (OSDM) having greater HDL-SD. The follow-up time was significantly (*P* < 0.05) different between the UA-SD and TG-SD groups, with patients who underwent follow-up for 1–3 years exhibiting the lowest UA-SD and TG-SD. See Table 2 for more details.

**Table 2:** Analysis of factors influencing the variability in risk factors for diabetes mellitus.

Variable	UA-SD			TG-SD			TC-SD			HDL-SD			LDL-SD		
	Mean (SD)	Med (IQR)	Effect size	Mean (SD)	Med (IQR)	Effect size	Mean (SD)	Med (IQR)	Effect size	Mean (SD)	Med (IQR)	Effect size	Mean (SD)	Med (IQR)	Effect size
Sex			2.05			− 0.66			− 1.03			− 3.35			− 0.86
Male	50.84 ± 31.11	44.41(32.40,59.69)	*P* = 0.041	0.84 ± 1.48	0.32(0.20,0.74)	*P* = 0.509	0.59 ± 0.36	0.55(0.35,0.76)	*P* = 0.302	0.47 ± 4.46	0.13(0.09,0.16)	*P* < 0.001	0.52 ± 0.61	0.47(0.30,0.62)	*P* = 0.389
Female	44.29 ± 30.05	35.75(25.79,53.41)		0.73 ± 1.72	0.34(0.20,0.58)		0.64 ± 0.50	0.54(0.38,0.78)		0.17 ± 0.10	0.14(0.11,0.19)		0.53 ± 0.35	0.47(0.30,0.68)	
Age, years			− 0.45			− 2.88			2.37			− 1.41			− 1.45
≤ 60	46.92 ± 25.06	40.69(30.20,56.67)	*P* = 0.652	0.98 ± 1.63	0.36(0.20,0.88)	*P* = 0.004	0.67 ± 0.54	0.59(0.39,0.82)	*P* = 0.019	0.15 ± 0.09	0.13(0.09,0.17)	*P* = 0.158	0.57 ± 0.65	0.48(0.29,0.67)	*P* = 0.146
> 60	48.37 ± 35.14	40.52(28.15,54.76)		0.61 ± 1.55	0.29(0.19,0.55)		0.56 ± 0.30	0.51(0.35,0.74)		0.48 ± 4.42	0.14(0.10,0.17)		0.49 ± 0.29	0.42(0.30,0.61)	
OSDM			− 1.45			− 1.60			− 1.64			− 2.01			− 1.17
Yes	46.04 ± 28.53	39.95(29.36,54.66)	*P* = 0.149	0.63 ± 1.04	0.32(0.20,0.60)	*P* = 0.110	0.59 ± 0.37	0.54(0.37,0.72)	*P* = 0.104	0.40 ± 3.82	0.14(0.10,0.18)	*P* = 0.044	0.49 ± 0.29	0.47(0.30,0.63)	*P* = 0.244
No	51.56 ± 35.25	43.31(29.07,64.82)		1.15 ± 2.42	0.35(0.20,0.85)		0.68 ± 0.55	0.58(0.33,0.89)		0.14 ± 0.08	0.13(0.09,0.17)		0.61 ± 0.79	0.49(0.31,0.71)	
Follow-up			9.97			7.36			1.07			5.38			5.91
1–3 years	37.14 ± 23.24^a,b^	31.98(22.62,48.35)	*P* < 0.001	0.74 ± 1.46	0.26(0.16,0.59)^c^	*P* = 0.025	0.57 ± 0.38	0.53(0.28,0.78)	*P* = 0.345	0.15 ± 0.10	0.13(0.09,0.17)	*P* = 0.068	0.46 ± 0.27	0.42(0.23,0.64)	*P* = 0.052
4–6 years	51.44 ± 34.55^a^	40.73(31.60,63.66)		0.76 ± 1.91	0.34(0.20,0.60)		0.62 ± 0.48	0.53(0.38,0.74)		0.14 ± 0.07	0.13(0.10,0.18		0.51 ± 0.27	0.46(0.31,0.64)	
≥ 7 years	52.88 ± 30.51^b^	45.86(36.08,61.88)		0.84 ± 1.38	0.36(0.23,0.72)^c^		0.65 ± 0.44	0.58(0.41,0.78)		0.64 ± 5.31	0.14(0.11,0.17)		0.60 ± 0.74	0.48(0.34,0.67)	

Type of effect size for UA-SD and TC-SD: independent samples t-test for Sex, Age, OSDM; one-way ANOVA for Follow-up time.

Type of effect size for TG-SD, HDL-SD, and LDL-SD: sex, age, and OSDM using Mann–Whitney U test; Follow-up time using Kruskal–Wallis test.

*OSDM* outpatient special disease management, *UA-SD* uric acid standard deviation, *TG-SD* triglyceride standard deviation, *TC-SD* total cholesterol standard deviation, *HDL-SD* high-density lipoprotein standard deviation, *LDL-SD* low-density lipoprotein standard deviation.

^a^There was a between group difference between 1–3 and 4–6 years of follow-up.

^b^There were between group difference at 1–3 years and ≥ 7 years of follow-up.

^c^There were between group difference at 1–3 years and ≥ 7 years of follow-up.

### Binary logistic regression analysis of the relationship between the variability in diabetes risk factors and complications

The variables of age, sex, outpatient special disease management (OSDM), follow-up time, UA-SD, TG-SD, TC-SD, HDL-SD, and LDL-SD were subjected to univariate logistic regression analysis. The analyses found that OSDM, TG-SD, and TC-SD were influential factors (*P* < 0.05) for the occurrence of complications and diabetic peripheral neuropathy; Age, TG-SD, and TC-SD were influential factors (*P* < 0.05) for diabetic peripheral vasculopathy (refer to the supporting information S1). And statistically significant variables (*P* < 0.05) were subsequently incorporated into a multivariate logistic regression model.

The outcomes demonstrated that OSDM had a protective effect against complications [OR = 0.53, 95% CI (0.29–0.10)], whereas TC-SD was identified as a risk factor for complications [OR = 2.42, 95% CI (1.18–4.97)] (Table 3). Moreover, OSDM had a protective effect against diabetic peripheral neuropathy [OR = 0.51, 95% CI (0.303–0.862)], while TC-SD was associated with an increased risk of diabetic peripheral neuropathy [OR = 2.29, 95% CI (1.17–4.50)] (Table 4). Additionally, TC-SD was identified as a risk factor for diabetic peripheral vascular disease [OR = 2.50, 95% CI (1.25–5.02)] (Table 5). Notably, the study included a limited number of patients with a history of diabetic nephropathy (9 participants) or retinopathy (6 participants), which did not meet the requirements for inclusion in the binary logistic regression analysis; therefore, these conditions were not considered in the model analysis.

**Table 3:** Multivariate logistic regression analysis of diabetic risk factor variability and the occurrence of complications.

Variable	*B*	S$\overline{\chi }$	Wals $x^{2}$	OR	95% CI		*P*
					Lower	Upper	
OSDM	− 0.66	0.31	4.14	0.53	0.29	0.98	0.042
TG-SD	0.09	0.08	1.12	1.09	0.93	1.29	0.290
TC-SD	0.88	0.37	5.78	2.42	1.18	4.97	0.016

*OSDM* outpatient special disease management, *TG-SD* triglyceride standard deviation, *TC-SD* total cholesterol standard deviation.

**Table 4:** Multivariate logistic regression analysis of diabetic risk factor variability and diabetic peripheral neuropathy incidence status.

Variable	*B*	S$\overline{\chi }$	Wals $x^{2}$	OR	95% CI		*P*
					Lower	Upper	
OSDM	− 0.67	0.27	6.34	0.51	0.30	0.86	0.012
TG-SD	0.04	0.08	0.25	1.04	0.89	1.22	0.619
TC-SD	0.83	0.34	5.81	2.29	1.17	4.50	0.016

*OSDM* outpatient special disease management, *TG-SD* triglyceride standard deviation, *TC-SD* total cholesterol standard deviation.

**Table 5:** Multivariate logistic regression analysis of diabetic risk factor variability and diabetic peripheral vasculopathy.

Variable	*B*	S$\overline{\chi }$	Wals $x^{2}$	OR	95% CI		*P*
					Lower	Upper	
Age	− 0.02	0.01	2.51	0.99	0.97	1.00	0.114
TG-SD	0.07	0.08	0.75	1.07	0.91	1.26	0.384
TC-SD	0.92	0.36	6.66	2.50	1.25	5.02	0.010

*TG-SD* triglyceride standard deviation, *TC-SD* total cholesterol standard deviation.

## Discussion

### Current status of complications in diabetic patients

Within the scope of this study, diabetic peripheral neuropathy and diabetic peripheral vasculopathy were the two complications with the highest incidence. Specifically, the incidence rate of diabetic peripheral neuropathy was 77.2%, similar to the findings reported by Su et al.^37^. The primary clinical manifestation of diabetic peripheral neuropathy involves symmetrical limb pain, particularly in the distal regions. In severe cases, it may be accompanied by infection resulting from foot ulceration or gangrene, thereby engendering the potential for amputation or disability. Such grave outcomes significantly jeopardize patients’ overall quality of life and physical and mental well-being^38^. Furthermore, the incidence rate of diabetic peripheral vascular disease was 62.6%, which was lower than that reported in the study conducted by Chiou et al.^39^. The primary pathological alterations associated with diabetic peripheral vascular disease include systemic atherosclerosis, thrombosis, plaque formation, and the occurrence of luminal stenosis and occlusion in select patients, ultimately culminating in distal limb ischaemia. This condition serves as an autonomous risk factor for diabetic foot complications and amputation^40^, concurrently serving as an indicative marker of systemic atherosclerosis and a potent predictor of cardiovascular ischaemic events^11^. This should draw clinical attention to the fact that the burden of T2DM complications is rising dramatically.

### Analysis of factors influencing the variability in risk factors for diabetes

The study findings revealed that sex significantly influences UA-SD, with men exhibiting greater UA-SD than women. This disparity can be attributed to the fact that men are generally shown to have greater uric acid levels than women. One investigation determined that serum uric acid levels were markedly greater in men than in women, and the incidence of hyperuricaemia was also significantly greater among men^41^. The lower serum uric acid levels observed in women can be attributed to higher plasma oestrogen concentrations in females, which leads to a notably diminished postsecretory reabsorption of urate by the renal tubules. Consequently, women exhibit heightened clearance of uric acid through the kidneys^42^. Furthermore, the duration of follow-up serves as an influential factor in UA-SD, whereby an extended follow-up period correlates with heightened fluctuations in uric acid levels. A prolonged follow-up duration reflects a longer disease course, and previous studies have revealed a positive association between the duration of type 2 diabetes and elevated HbA1c levels^43^. Glycosylated haemoglobin levels are linearly correlated with serum uric acid levels, and alterations in HbA1c levels result in greater fluctuations in serum uric acid levels^44^. Additionally, patients with longer follow-up durations exhibited a greater frequency of visits at which uric acid was measured, thereby providing a more comprehensive depiction of uric acid variability.

In this study, age was found to be a significant influencing factor for TG-SD and TC-SD, with patients aged ≤ 60 years exhibiting greater TG-SD and TC-SD. Previous studies have indicated^45^ that lipid profiles are predominantly affected in individuals within the age range of 20–60 years, with relatively diminished variations observed in individuals older than 60 years of age. Notably, TG and TC levels tend to peak between 50 and 59 years of age, while HDL-C and LDL-C levels exhibit their highest levels between 40 and 49 years of age^46^. It is plausible that diabetic patients younger than the age of 60 years exhibit a preference for a high-fat meat-based diet, which exerts a considerable influence on lipid levels^47^. This dietary approach contributes to heightened fluctuations in lipid levels and increased variability over time, particularly among individuals with elevated lipid levels^45^. Additionally, Tolassa et al.^48^ also revealed that young individuals tend to consume diets rich in meat and processed foods, which are predictive factors for abnormal lipid level elevations.

The results of this study showed that sex and outpatient-specific disease management were influencing factors for HDL-SD. HDL-SD was greater in women than in men in this study. Thelle et al.^49^ also reported that the mean HDL cholesterol level in women was greater than that in men, and the fluctuations in oestrogen levels in female patients can lead to fluctuations in HDL cholesterol^50^, which may be the reason for the greater variability in HDL cholesterol in female patients. In addition, related studies have shown^51^ that in women with T2DM, HDL cholesterol is a major predictor of bone changes and that changes in HDL cholesterol are associated with an increased risk of cardiovascular disease. HDL-SD levels were greater in patients without outpatient-specific disease management. This disparity can be attributed to the fact that patients receiving outpatient special disease management benefit from regular and structured follow-up appointments, which include glycosylated haemoglobin monitoring and routine screening for complications conducted every three months^52^. Such diligent monitoring helps patients manage their condition and promotes treatment compliance. Notably, the utilization rate of statin therapy is considerably greater among type 2 diabetes patients enrolled in outpatient special disease management than among those not under such care. The regular use of statins effectively reduces and stabilizes lipids in patients. Multiple studies have consistently affirmed that health insurance plays a pivotal role in improving medication standardization and enhancing the quality of care for individuals with chronic diseases, consequently contributing to improved health outcomes^52,53^.

### Effect of diabetes risk factor variability on complications

In this study, TC-SD was identified as a significant risk factor for the development of complications, specifically diabetic peripheral neuropathy and diabetic peripheral vasculopathy, which is in line with the findings of scholars such as Hukportie et al.^54^, who reported that cholesterol variability increases the risk of developing diabetic neuropathy. The pathogenesis of diabetic peripheral neuropathy is currently believed to be linked to insulin deficiency, insulin resistance, hyperglycaemia, and dyslipidaemia^55^. Dyslipidaemia contributes to elevated levels of oxidized LDL cholesterol and free fatty acids, culminating in increased production of inflammatory factors and heightened inflammatory response signalling, ultimately leading to vascular damage^9,56^. Prior research has also established a correlation between lipid variability and diabetic vascular complications^57^.

TC-SD serves as a risk factor for diabetic vasculopathy, and Waters et al.^58^ also found lipid variability to be a predictor of cardiovascular events in diabetic patients. It is widely recognized that lipid levels exhibit considerable variations among diabetic patients^59^. Another study demonstrated^60^ that individuals with diabetes exhibit a high incidence of vascular disease due to elevated levels of total plasma cholesterol, triglycerides, and dense lipoproteins, as well as a high sensitivity of platelets to aggregating agents and a hypercoagulable state. The impact of lipid variability on vascular complications is attributed to induced oxidative stress, where substantial fluctuations in lipids can destabilize plaques, leading to the release of atherogenic substances. The vascular endothelium sustains greater damage, thereby increasing the likelihood of developing diabetic vascular disease^61^.

The influence of risk factors and their variability on diabetic complications should not be underestimated. Long-term variability in total cholesterol levels serves as a predictor of diabetic peripheral neuropathy and diabetic peripheral vasculopathy. The increased lipid fluctuations observed in diabetic patients under the age of 60 emphasize the importance of diligently managing lipid levels in the daily treatment of diabetes, particularly in this age group. Regular monitoring of lipid levels and timely pharmacological intervention should be provided for patients with abnormal or substantially variable lipid profiles. However, there is currently a lack of relevant studies establishing a direct causal relationship between long-term variability in total cholesterol levels and diabetic complications, although emerging evidence indicates that variability in diabetic risk factors is indeed associated with complications, necessitating further research.

The findings of this study demonstrated that outpatient special disease management serves as a protective factor against the progression of complications and diabetic peripheral neuropathy. Consistent with the findings of Lewing et al.^62^, the utilization of primary health care for diabetes was associated with complications. The consistent use of statins and glucose-lowering medications among patients receiving outpatient special disease management, coupled with regular monitoring and screening for complications, may represent an important aspect of outpatient special disease management in safeguarding against the development of complications in patients with diabetes. One study^63^ has shown that enhanced management and integration of individuals with chronic conditions within primary care settings yield improved health outcomes and cost savings. Notably, outpatient special disease management for patients with diabetes offers comprehensive testing, follow-up care, and disease management^64^. These practices contribute to better glycaemic control and reduced lipid fluctuations, subsequently mitigating the occurrence of unfavourable outcomes^65^.

However, this study did not establish a correlation between variability in risk factors and the onset of diabetic nephropathy or diabetic retinopathy, possibly due to the limited number of patients with those complications, which constitutes one of the limitations of this study. Furthermore, the study revealed that lipid levels and variability were lower in patients receiving special disease management for diabetes than in those not receiving such services. This observation underscores the impact of patient treatment adherence on lipid profiles. On the one hand, while managing patients via outpatient special disease management, health care providers can enhance public awareness to encourage diabetic individuals to enrol in such programs. Research has emphasized^66^ that outpatient special disease management can enhance patients' adherence to medical treatment and can regularly provide patients with disease knowledge guidance. Guidance related to chronic disease rehabilitation and relevant lifestyle recommendations are essential measures for maintaining disease stability and preventing complications^67^. On the other hand, for patients without outpatient special management, additional guidance on dietary choices, exercise regimens, and medication adherence should be provided during their visits. This approach aids in blood glucose control, lipid stabilization, and delay of complication occurrence and progression. Clinical staff involved in the care of diabetic patients should adhere to established standards of care for type 2 diabetes mellitus, promptly detecting and diligently monitoring diabetic complications.

This study has several limitations. First, it should be noted that this investigation is confined to a single-centre setting, resulting in a limited number of positive outcomes for certain complications. Consequently, this circumstance introduces a certain degree of bias to the results and may impede the generalizability of the findings. Furthermore, the absence of data concerning the duration and dosage of statin therapy within this study precludes the assessment of the impact of statin therapy on lipid variability. Subsequent research endeavours are warranted to elucidate the influence of lipid-lowering medications on lipid variability. The duration of the patients' illnesses was also not recorded, but the follow-up time was used instead, which may have biased the results of the study. Finally, this study did not explore the role of medications in complication incidence because the investigators considered that the effect of medications on complications is susceptible to differences in clinical treatment regimens, patient conditions, individual characteristics, medication adherence, and other factors; which biased the data collection and may have affected the results of this retrospective study to some extent. In future studies, it is recommended that multicentre explorations be conducted to include more diabetic patients with complications, and the effects of medications on the development of complications can be explored through a prospective design with further exploration of the causality and mechanism of risk factor variability and diabetic complications.

## Conclusion

In this study, TC-SD was found to be a risk factor for the occurrence of diabetic peripheral neuropathy and diabetic peripheral vasculopathy, whereas outpatient special disease management functions as a protective factor against complications and diabetic peripheral neuropathy. In the comprehensive management of diabetic patients over the long term, regular assessment of lipid levels should be incorporated alongside routine blood glucose monitoring. By expanding the accessibility of the medical insurance system to include outpatient special disease management for patients with diabetes, patients can improve their adherence to treatment and follow-up to control their blood lipid levels in a normal and stable state, thereby slowing the progression of diabetic complications, improving patients' overall survival and quality of life, and alleviating the economic burdens faced by families and society.

### Supplementary Information

Supplementary Tables.

## Data Availability

The datasets generated and/or analysed during the current study are available from the corresponding author upon reasonable request.
