# Supplementary material for: The association between variability of risk factors and complications in type 2 diabetes mellitus: a retrospective study
Source: Sci Rep. 2024 Mar 16;14:6357. doi: 10.1038/s41598-024-56777-w (PMC10943073; doi:10.1038/s41598-024-56777-w)
Supplement: Supplementary file 1 — Supplementary Tables. [file 41598_2024_56777_MOESM1_ESM.docx]

**Supporting Information**

Table 1 Single-Factor logistic regression analysis of diabetic risk factor variability and the occurrence of complications

| Variable | *B* | S$\overline{\chi}$ | Wals $x^{2}$ | *P* | OR（95%CI） |
| --- | --- | --- | --- | --- | --- |
| Sex | -0.098 | 0.291 | 0.114 | 0.735 | 0.906（0.513~1.603） |
| Age | -0.023 | 0.012 | 3.376 | 0.066 | 0.977（0.954~1.002） |
| OSDM | -0.772 | 0.297 | 6.734 | 0.009 | 0.462（0.258~0.828） |
| Follow-up time | -0.067 | 0.065 | 1.075 | 0.300 | 0.935（0.824~1.062） |
| UA-SD | 0.005 | 0.004 | 1.117 | 0.291 | 1.005（0.996~1.013） |
| TG-SD | 0.238 | 0.080 | 8.847 | 0.003 | 1.268（1.084~1.484） |
| TC-SD | 1.155 | 0.333 | 12.034 | ＜0.001 | 3.175（1.653~6.099） |
| HDL-SD | -0.044 | 0.148 | 0.088 | 0.767 | 0.957（0.715~1.280） |
| LDL-SD | 0.118 | 0.245 | 0.232 | 0.630 | 1.125（0.696~1.817） |

OSDM：Outpatient special disease management; UA-SD：Uric acid standard deviation;

TG-SD：Triglycerides standard deviation; TC-SD：Total cholesterol standard

deviation; HDL-SD：High-density lipoprotein standard deviation;

LDL-SD：Low-density lipoprotein standard deviation

Table 2 Single-Factor logistic regression analysis of diabetic risk factor variability and diabetic peripheral neuropathy

| Variable | *B* | S$\overline{\chi}$ | Wals $x^{2}$ | *P* | OR（95%CI） |
| --- | --- | --- | --- | --- | --- |
| Sex | -0.108 | 0.249 | 0.188 | 0.664 | 0.898（0.551~1.462） |
| Age | -0.015 | 0.011 | 1.863 | 0.172 | 0.986（0.965~1.006） |
| OSDM | -0.760 | 0.259 | 8.641 | 0.003 | 0.468（0.282~0.776） |
| Follow-up time | -0.022 | 0.055 | 0.160 | 0.689 | 0.978（0.878~1.089） |
| UA-SD | 0.003 | 0.004 | 0.421 | 0.517 | 1.003（0.995~1.010） |
| TG-SD | 0.178 | 0.075 | 5.690 | 0.017 | 1.195（1.032~1.383） |
| TC-SD | 0.998 | 0.309 | 10.233 | 0.001 | 2.686（1.466~4.920） |
| HDL-SD | -0.071 | 0.253 | 0.079 | 0.779 | 0.931（0.567~1.530） |
| LDL-SD | 0.066 | 0.231 | 0.080 | 0.777 | 1.068（0.678~1.681） |

OSDM：Outpatient special disease management; UA-SD：Uric acid standard deviation;

TG-SD：Triglycerides standard deviation; TC-SD：Total cholesterol standard

deviation; HDL-SD：High-density lipoprotein standard deviation;

LDL-SD：Low-density lipoprotein standard deviation

Table3 Single-Factor logistic regression analysis of diabetic risk factor variability and diabetic peripheral vasculopathy

| Variable | *B* | S$\overline{\chi}$ | Wals $x^{2}$ | *P* | OR（95%CI） |
| --- | --- | --- | --- | --- | --- |
| Sex | -0.044 | 0.215 | 0.041 | 0.839 | 0.957（0.628~1.460） |
| Age | -0.021 | 0.009 | 4.849 | 0.028 | 0.980（0.962~0.998） |
| OSDM | -0.320 | 0.233 | 1.894 | 0.169 | 0.726（0.460~1.145） |
| Follow-up time | -0.019 | 0.048 | 0.152 | 0.697 | 0.982（0.894~1.078） |
| UA-SD | 0.004 | 0.003 | 1.445 | 0.229 | 1.004（0.997~1.011） |
| TG-SD | 0.209 | 0.084 | 6.141 | 0.013 | 1.233（1.045~1.454） |
| TC-SD | 1.100 | 0.324 | 11.519 | ＜0.001 | 3.004（1.592~5.670） |
| HDL-SD | 0.174 | 0.612 | 0.081 | 0.776 | 1.190（0.359~3.944） |
| LDL-SD | 0.269 | 0.247 | 1.186 | 0.276 | 1.308（0.807~2） |

OSDM：Outpatient special disease management; UA-SD：Uric acid standard deviation;

TG-SD：Triglycerides standard deviation; TC-SD：Total cholesterol standard

deviation; HDL-SD：High-density lipoprotein standard deviation;

LDL-SD：Low-density lipoprotein standard deviation
